# Supplementary material for: Perceived listening ability and hearing loss: Systematic review and qualitative meta-synthesis
Source: PLoS One. 2022 Oct 25;17(10):e0276265. doi: 10.1371/journal.pone.0276265 (PMC9595527; doi:10.1371/journal.pone.0276265)
Supplement: S1 Table — (DOCX) [file pone.0276265.s002.docx]

Hughes, SE, Boisvert, I, McMahon, CM, Steyns, A, Neal, K. Perceived listening ability and hearing loss: systematic review and qualitative meta-synthesis. PLOS One.

**S1 Table . Characteristics of included studies (N = 46)**

| **Study ID** | **Country** | **Participant characteristics** | | | | | | | **Aims** | **Data collection method** | **Data analysis method** |
| --- | --- | --- | --- | --- | --- | --- | --- | --- | --- | --- | --- |
|  |  | **N** | **Age**  **(Mean, SD)** | **Gender**  **(% female)** | **Ethnicity** | **Severity of HL** | **Device use** | **Comorbid-ities** |  |  |  |
| Alsawy 2020[38] | UK | 9 | 76.4 years (range = 54 - 86) | 4 (44.4%) | 88.9% White British; 11.1% Mixed | N/A | N/A | Dementia, "other comorbid health problems" | To explore what makes communication with their family carers meaningful from the perspective of people living with dementia. | Video reflective tool, semi-structured interviews | Thematic analysis |
| Athalye 2014[39] | UK | 10 | 56.1 years (range 25-79) | 3  30% | Not reported | Severe-profound SNHL | unilateral HA = 2, bilateral HAs = 4, BAHA = 1, CI = 2, none = 1 | Not reported | Experiences of adults assessed for CI but did not proceed to implantation | Interviews | Thematic content analysis |
| Bennett  2021[40] | Australia | 30 | Range 19 - 88 | 21 (70.0%) | Not reported | Not reported | HAs = 17; CI+CI = 2; unaided = 2 | Not reported | To explore the lived experience of social challenges and emotional distress in relation to HL and the coping mechanisms employed to manage them. | Semi-structured interviews and focus groups | Thematic analysis |
| Bennion 2013[41] | UK | 9 | 79.9 years (range 61 - 93) n = 8 | 6 66.6% | Not reported | Not reported | Bilateral hearing aids = 5; unilateral HA = 4 | Not reported | To explore, and develop a greater understanding of the experience of living with age-related hearing impairment from the perspectives of older people themselves & highlight possible recommendations to improve hearing aid (HA) services and rehabilitation | Semi-structured interview | Thematic analysis |
| Bryant  2020[42] | Australia | 14 | 70.5 (SD = 4.45, range = 64 – 80) | 6 (42.9%) | Not reported | Not reported | HAs = 10, HA+CI = 4 | Depress-ion, anxiety, psychosis | "To understand experiences of HL and audiological rehabilitation from the perspective of older adults with comorbid psychological symptoms (e.g., depression, anxiety, psychosis)." | Semi-structured interviews | Thematic analysis |
| Choi 2018[43] | USA | 19 | Not reported | Not reported | Asian | None - Moderate | None | Not reported | To explore experiences related to HL and barriers to hearing health care among older Korean Americans | Focus groups, interviews | Inductive content analysis |
| Cudmore 2017[44] | Ireland | 100 | 73 (SD = 0.9) | 39 (39%) | Not reported | 57% HL (21% unilateral, 36 % bilateral), 43% WNL | Not reported | Not reported | “To calculate baseline prevalence of communication breakdown and to explore communication breakdown with older people with HL during clinical consultations.” | Semi-structured interview | Thematic analysis |
| Davies 2001[45] | UK | 207 | > 55 years) | 157 75.8% | Not reported | Self-report | Not reported | Not reported | To determine the extent to which acoustic problems in the built environment affect elderly people. | Survey, diary log of activity, interviews, focus groups | Analysis method not reported, analysis of textual data |
| Davis 2021[46] | USA | 43 | 53.5 years (SD 16.2, range 20-77 years) | 32 | Not reported | Mean PTA = 42.4 db HL in better ear | Mixed (HA = 34; CI = 3; non-users = 6) | Not applicable | “To identify key domains and constructs of listening-related fatigue”...”to create a theoretical framework for understanding listening-related fatigue that could guide the development of a reliable and valid assessment tool.” | Focus groups | Thematic analysis |
| Dawes 2014[47] | UK | 16 | 72.9 years | 7 (43.8%) | Not reported | mean = 22 db HL for 250 - 1kHz; mean = 56 db HL for 2kHz - 8kHz) = MMHL | HAs | Not reported | “To describe getting used to hearing aids from the perspective of adult hearing aid users” | Focus groups | Content analysis |
| Fitzpatrick 2010[48] | Canada | 14 | Range 48 - 71 years | 10 71.4% | Not reported | Profound (mean PTA CI ear = 98 dB HL; mean PTA contralateral ear = 103 dB HL) | CI | Not reported | To explore the benefits of an FM system in real-world environments from the perspective of adults with cochlear implants and to explore the factors and barriers to using an FM system with a cochlear implant | Diary and question-naire | Thematic analysis |
| Foster 2003[49] | USA | 15 | Not reported | not reported | Not reported | Not reported | Not reported | Not reported | “To describe the experiences of deaf professionals who have succeeded in attaining supervisory roles in work environments where they were the only one of very few deaf employees.” | Semi-structured interviews | Ethno-graphy |
| Fulford 2011[50] | UK | 12 | Range 17 - 72 years | 9  75% | Not reported | Mild to profound | None or HAs | Not reported | To find out more about how, why, and in what social contexts people with hearing impairment make music | Interviews | Thematic network analysis |
| Funk 2018[51] | USA | 8 | 80.5 years (range = 70 - 95 years) | 3  37.5% | Caucasian | Self-reported, Severity not reported | Not reported | Not reported, hospital-ised | To assess the hospital experience of older adults with hearing impairment to formulate suggestions for improving nursing care. | Interviews | Thematic analysis |
| Gfeller 2019[52] | USA | 40 | Range = 19 - 81 years | Not reported | Not reported | Not reported | CI, EAS, SSD | Not reported | “To explore the perspectives of adult CI recipients regarding two experiences with music in everyday life: purposeful music listening and background music that competes with spoken conversation. To develop a framework of everyday music experiences based upon CI perspectives that could inform future rehabilitative practices and research initiatives.” | Online question-naire | Grounded theory |
| Giolas 1967[53] | USA | 20 | Not reported (> 14 yrs) | Not reported | Not reported | Unilateral (PTA > 40 dB) |  | Not reported | “To learn more about the specific nature of the problems associated with monaural hearing.” | Interviews | Critical incident technique |
| Granberg 2014[54] | Sweden | 36 (Dutch = 16; SA = 20) | Dutch: range = 29 - 87 years; SA: range = 25 - 84 years | Dutch: 56%; SA: 35% | Not reported | Mild to profound | Not reported | Not reported | “To explore areas of functioning, disability, and environmental factors of adults with HL (HL) by using the ICF classification.” | Focus groups | Content analysis |
| Hallam 2008[55] | UK | 25 | 53.4 years (SD = 13.0, range = 25-77) | 14  56% | Not reported | Profound | HA = 12 (48%);  CI = 10 (40%) | Not reported | To examine the impact of acquired profound HL (APHL) on the relationship between the hearing-impaired person and their normally hearing close family member, and to identify the kinds of adjustment leading to maintenance or deterioration of the relationship. | Interviews | Grounded theory |
| Hallberg 1991[56] | Sweden | 62 | 53 years | 25  40.3% | Not Stated | SNHL and CHL  PTA >30 dB HL | HAs  (n = 27) | Not reported | To gain a deeper understanding of what hearing-impaired people are doing to deal with demanding auditory situations in everyday life. | Interviews | Grounded theory |
| Hallberg 1993[57] | Sweden | 10 | 52.5 years (range = 45 - 58) | 10  100% | Not reported | Severe | N/A | Not reported | “To describe, from the perspective of the spouses, their experience of living close to a male with severe noise-induced HL; to generate the first step of a theory of how these spouses manage their daily life.” | Interviews | Grounded theory |
| Hallberg 1995 | Sweden | 53 | 54.8 years  (SD = 3.4 years) | 0 (0%) | Not reported | Noise-induced HL (PTA = 35 dB HL) | Not reported | Not reported | “To describe coping with noise-induced HL from the perspective of middle-aged men; to gain a deeper insight in their experiences of suffering from NIHL.” | Interviews | Grounded theory |
| Hallberg 1996[58] | Sweden | 10 | 59 years (SD = 7.6, range 46 - 71) | 10  100% | Not reported | PTA = 59.6 dB HL | HA | Not reported | “To describe, from the perspective of women with noise-induced HL (NIHL), their experiences of noise as a threat to health and their having to live with a hearing disability.” | Interviews | Grounded theory |
| Hass-Slavin 2005[59] | Canada | 13 | Not reported | 3 (23.1%) | Not reported | Self-reported HL in at least one ear | Not reported | Not reported | “To explore the challenges and coping strategies experienced by dairy farmers with self-reported HL and communication difficulties.” | In-depth interviews | “a process that results in the fundamental tree structure” |
| Heacock 2019[60] | USA | 12 | Range = 22 - 58 years | 8 (66.7%) | Not reported | N/A | N/A | Not reported | “To describe the role of the adult child in the hearing healthcare process of a parent with HL.” | Semi-structured interview | Thematic analysis |
| Hua 2015[61] | Sweden | 15 | 47.4 years  (SD = 14.0, range = 18 - 65 years) | Not reported | Not reported | Mild-moderate | HAs | Not reported | “To explore the conceptions of working life among employees with mild-moderate aided hearing impairment.” | Semi-structured interviews | Phenomeno-graphy |
| Hughes 2018[62] | UK | 17 | 64.1 years (range 42 - 84) | 8  47.2% | 16 White British (94.1%) | SPHL | HA, CI+HA,  CI | Not reported | To explore the perceptions, understanding, and experiences of listening effort in adults with severe-profound sensorineural HL before and after cochlear implantation | Focus groups | Grounded theory |
| Iezzoni 2004[63] | USA | 26 | Deaf adults (range = 23 - 51 years); HOH (range 30 - 74 years) | 14 (53.8%) | White = 22 (96.2%); Non-white or Hispanic = 4 (3.8%) | D/deaf & HOH | HA (n = 18) | Not reported | “To understand perceptions of health care experiences and suggestions for improving care among deaf or hard-of-hearing individuals.” | Semi-structured group interviews | Not specified |
| Jeffs 2015[64] | UK | 8 | range = 20 – 49 years | Not reported | Not reported | Severe-profound SNHL | CI | Not reported | To explore the experiences of congenitally or early profoundly deafened candidates who receive cochlear implants as adults. | Semi-structured interviews | Grounded theory |
| Jonsson 2018[65] | Sweden | 10 | 47.3 years (range = 36 - 56 years) | 10 (100%) | Not reported | moderate HL | Not reported | Psoriasis, arthrosis, rheumatic, diabetes, heart, tinnitus | To achieve a deeper understanding of what a moderate HL means for middle-aged women. | Interviews | Phenomenology |
| Laplante-Levesque 2006[66] | Canada | 3 | 62, 69, 73 | 2  (66.7%) | Not reported | Mild-to-severe SNHL | HA | Not reported | To explore how an e-health approach could be used to facilitate communication between anew hearing aid user and the audiologist.” | Interviews, emails, audio-logical files | Grounded theory |
| Lockey 2010[67] | Canada | 4 | (range = 60 - 85 years) | 4  (100%) | Not reported | Mild to moderate-severe HL | HAs | Not reported | “To explore experiences surrounding hearing aid use and non-use in older women with HL to gain an in-depth understanding of the meaning and contextual issues that impact upon the adaptation process of older adults and their transitions in using hearing devices. | Interviews using biographic-narrative interview framework | Thematic analysis |
| Lucas 2018[68] | UK | 8 | 57.3 years  (range = 40 - 71) | 4  (50%) | Not reported | SSD | 1 = HA, 1 = CROS,  6 = none | Not reported | To examine the subjective psychological and social effects of highly asymmetric HL in adults (SSD) | Group interviews using the critical incident technique | Thematic analysis |
| McRackan 2017[69] | USA | 23 | 68.1 years (SD = 10.5, range = 46.2 - 84.2) | 10  (43%) | African American = 2 (9%); White = 21 (91%) | Severe-profound | CI | Not reported | To develop a CI-specific QOL instrument for adults | Focus groups | Grounded theory |
| Miller 2017[70] | USA | 17 | range = 26 - 89 years) | 10  (56%) | Not reported | None to severe HL (PTA range = 8 - 85 dB HL) | HAs = 71% of sample | Not reported | To evaluate the potential relevance of CPIB items for individuals with HL | Cognitive interviews + focus group with audio-logists (n = 3) | Descriptive quantitative analysis, (frequency counts), description of "trends" |
| Preminger 2014[71] | USA | 34 | range = 26 - 96 years | 19  (55.9%) | Nationality: Australian, Danish, American, British | Range PTA = 30.5 - 49.9 dB HL | HAs = 17 | Not reported | “To explore the perspectives of adults with hearing impairment on hearing help-seeking and rehabilitation.” | Multi-country, in-depth semi-structured interviews | Content analysis + inter-pretative phenomen-ology |
| Pryce 2012[72] | UK | 18 residents + 7 staff | 88.9 years (range = 75 - 99) | 14 (77.8%) | Not reported | Self-reported | HAs | Dementia (various types) | To explore the factors affecting communicating with a HL in residential care. | Observation, in-depth interviews | Ethno-graphy |
| Punch 2019[73] | USA | 8 | Not reported | Not reported | Not reported | Varied (range = mild to profound) | HAs = 6; CI = 1, CI+CI = 1 | Not reported | To identify multidimensional factors associated with health-related quality of life (HRQoL) for use in developing an inventory in which HRQoL is a core concept in evaluating the impact of HL and the efficacy of rehabilitative interventions | Focus groups using critical incident technique | Content analysis |
| Rembar 2009[74] | Norway | 74 | 56.2 years (SD = 15.2, range = 22 -81) | 44 (59%) | Not reported | severe-profound SNHL | CI | Not reported | To gain a deeper insight into the effects of cochlear implants on recipients’ lives, as perceived by the recipients themselves. | Open-ended question-naire | Descriptive analysis |
| Scarinci 2008[75] | Australia | 10 | 70.3 years (range = 60 - 83 years) | 5 (50%) | Not reported | Partner = HL range = mild to moderate-severe | Various | Not reported | To identify the International Classification of Functioning, Disability and Health (ICF) domains and categories that describe third-party disability of spouses of older people with hearing impairment. | In-depth interviews | Phenomen-ology |
| Scarinci 2009[76] | Australia | 10 | 70.2 years (SD = 7.12, range = 60 - 83) | 5  (50%) | Not reported | Partner's PTA = 41 dB HL (SD = 6.61, range = 32.5 - 50 dB HL) | Partner: HA+HA = 4 (40%), 1 x HA = 2 (20%), none = 4 (40%) | Not reported | “To identify the International Classification of Functioning, Disability and Health (ICF) domains and categories that describe third-party disability of spouses of older people with hearing impairment.” | Semi-structured interviews | Hermen-eutic phenomen-ological method-ology and the selective approach |
| Scharp 2020[77] | USA | 30 | 62.0 years (SD = 18.1, range = 20 - 90) | 20 (66.7%) | Not Hispanic or Latino = 29, Hispanic/ Latino = 1 | Mild to profound | At least 1 HA for each subject | Not reported | “To interrogate the social structures that promote and resist HL and hearing aid-related stigma. Specifically, to explore the ways the meaning of HL is constructed.” | Narrative interviews + semi-structured interviews | Contra-puntal analysis with thematic analysis |
| Schlau 2004[78] | USA | 24 | 53 years (range = 30 - 81) | 14 (58.3%) | White | Not reported | CI = 9 | Psychiatric disorder (n = 1) | “To examine the stories of those who have lived through acquired deafness to find patterns and discover a theory of adjustment to deafness, grounded in participants lived experiences.” | Semi-structured survey | Grounded theory |
| Shaw 2013[79] | Canada | 7 | range = 50 - 61 years | Not reported | Not reported | Mild to profound | HAs = 6, CI = 1 | Not reported | “To elicit the voices of persons with HL on the experiences, actions, processes, and strategies used to navigate accommodations in the work-place and to manage to live and work with HL.” | Interviews | Grounded theory |
| Tye-Murray 2009[80] | USA | 48 | 61 years (range = 29 - 79 years) | 21 (43.8%) | Not reported | Mean PTA = 51 dB HL better ear; 61 dB HL worse ear | HAs or CIs | Not reported | To gauge how HL affects the self-perceived job performance and psycho-emotional status of professionals in the workforce and to develop a profile of their aural rehabilitation needs. | Focus groups + workplace question-naire | Thematic analysis |
| Vaisberg 2019[81] | Canada | 12 | 67.8 years (SD = 9.5, range = 55 - 83) | 5  (41.7%) | Not reported | Mild to severe | HAs | Not reported | To explore the challenges that adult HA-wearing instrumentalists face, which prevent them from listening, responding to, and performing music. | Semi-structured interviews | Content analysis |
| Vieira 2018[82] | Brazil | 16 | 41.7 years (range = 28 - 58) | 5  (31.3%) | Not reported | severe-profound SNHL | Unilateral CI = 13 | Not reported | To understand the benefits of cochlear implantation in adulthood from the perspective of users. | Semi-structured interview | Grounded theory |
