# Supplementary material for: Perceived listening ability and hearing loss: Systematic review and qualitative meta-synthesis
Source: PLoS One. 2022 Oct 25;17(10):e0276265. doi: 10.1371/journal.pone.0276265 (PMC9595527; doi:10.1371/journal.pone.0276265)
Supplement: S1 File — (DOCX) [file pone.0276265.s004.docx]

Hughes, SE, Boisvert, I, McMahon, CM, Steyns, A, Neal, K. Perceived listening ability and hearing loss: systematic review and qualitative meta-synthesis. PLOS One.

S1 File. Medline Search Strategy.

| 1 | ((communication or listen* or auditory or aural or spoken or speech*OR heaing or listen*) adj3 (function* or strateg* or tactic* or breakdown or repair or percept*)).ti,ab. |
| --- | --- |
|  |  |
| 2 | auditory perception/ |
|  |  |
| 3 | Speech Perception/ |
|  |  |
| 4 | Communication/ |
|  |  |
| 5 | or/1-4 |
|  |  |
| 6 | deaf*.ti,ab. |
|  |  |
| 7 | (hearing aid* or cochlear or implant*).ti,ab. |
|  |  |
| 8 | (hearing adj3 (loss or impair* or disorder)).ti,ab. |
|  |  |
| 9 | Hearing Loss/ |
|  |  |
| 10 | Deafness/ or Cochlear Implants/ or Persons with Hearing Impairments/ |
|  |  |
| 11 | or/6-10 |
|  |  |
| 12 | (Qualitat* adj2 (research or method*)).ab,ti. |
|  |  |
| 13 | ((Grounded adj3 theory) or mix method* or phenomenolog* or ethnograph* or interview* or focus group* or (thematic adj3 analys*)).ab,ti. |
|  |  |
| 14 | Grounded Theory/ |
|  |  |
| 15 | Interview, Psychological/ or Interview/ |
|  |  |
| 16 | Focus Groups/ |
|  |  |
| 17 | ((("semi-structured" or semistructured or unstructured or informal or "in-depth" or indepth or "face-to-face" or structured or guide) adj3 (interview* or discussion* or questionnaire*)) or (focus group* or qualitative or ethnograph* or fieldwork or "field work" or "key informant")).ti,ab. |
|  |  |
| 18 | or/12-17 |
|  |  |
| 19 | 5 and 11 and 18 |
|  |  |
|  |  |
|  |  |
