# Supplementary material for: Perceived listening ability and hearing loss: Systematic review and qualitative meta-synthesis
Source: PLoS One. 2022 Oct 25;17(10):e0276265. doi: 10.1371/journal.pone.0276265 (PMC9595527; doi:10.1371/journal.pone.0276265)
Supplement: Attachment — Submitted filename: PlosOne_YK_Reviewcomments_25July2022.docx [file pone.0276265.s006.docx]

It was a good reading to review “Perceived listening ability and hearing loss: systematic review and qualitative metasynthesis”. It was well thought study, especially when many studies have been done in this area across the globe. The study can be considered for publication with minor revisions.

The researchers have selected a good topic and the fact of the number of articles they have included justifies the need for a systematic review with metasynthesis. Have done good effort on including relevant studies and search engines. The method adapted justifies the research question and well explained. Author may give explanation on the need to include Google scholar and specifically only first 200 records). If the editor justifies the need to be included that comment in the manuscript that is fine, if not, I am ok to seek clarification.

In the results section, the authors have made a good attempt to summarize and present in well structured sentences. However, in table 3 the author has given quality appraisal of the articles. It is observed that, for the 6^th^ question, on relationship between researcher and participants many studies the relation was not clear. In a research this relation is important. May be good idea if the author, although mentioned, good to comment how this can influence on the interpretation of the results. In the themes table 4, may be good to include the codes instead of bullets to get more clarity.

It is also appreciated the author has presented the discussion well and highlighted the limitations of the study. It will be good the implication/conclusions/themes drawn in the study are taken further.
